# Two Cases of Idiopathic Condylar Resorption Due to Temporomandibular Joint (TMJ) Osteoarthritis Remodeled by Different Treatment Modalities

**DOI:** 10.7759/cureus.49322

**Published:** 2023-11-24

**Authors:** Syuntaro Nomoto, Takuya Ota, Hideshi Sekine

**Affiliations:** 1 Department of Fixed Prosthodontics, Tokyo Dental College, Tokyo, JPN

**Keywords:** anchor screws, occlusal splint, open bite, temporomandibular joint, condylar resorption

## Abstract

Condylar resorption is a condition of progressive and significant mandibular head resorption. We treated two patients with condylar resorption caused by temporomandibular joint (TMJ) osteoarthritis. The first patient was a 22-year-old female at the time of the initial examination. She had a history of orthodontic treatment and came to the clinic with a number of symptoms, including difficulty opening the mouth. Idiopathic condylar resorption was diagnosed, an occlusal splint was placed for approximately one year, and the patient was followed up for 12 years. The second patient was a 20-year-old female who had completed non-extraction orthodontic treatment by the age of 17 years. She came to the clinic with esthetic issues, such as an anterior open bite. Implant anchor screws were placed in the alveolar bone of the anterior teeth and intermaxillary fixation was performed. Case 1 had 12 years of follow-up, with CT scan results showing remodeling of the condylar. Case 2 is expected to shorten treatment time. However, the CT scan showed remodeling and improvement in the chief complaint.

## Introduction

Idiopathic condylar resorption (ICR) is defined as specific progressive resorption of the mandible accompanied by a marked decrease in the height of the mandibular ramus. Shortening of the mandibular branch height due to ICR may cause mandibular retraction or an open bite. Cases with a similar pathology to ICR and markedly progressive mandibular head resorption are classified as progressive condylar resorption (PCR) [1]. Temporomandibular joint osteoarthritis (TMJ-OA) is one of the factors contributing to the pathogenesis of ICR/PCR [1,2]. TMJ-OA is a degenerative joint disease that is characterized by progressive cartilage deterioration, subchondral bone remodeling, and chronic inflammation in synovial tissue [3]. It is said that the release of mechanical stress leads to an improvement in ICR. I believe that there are few reports on treatment with splints with a 12-year follow-up. As for treatment with anchor screws, not much is known to the world and the duration of treatment is short.

In clinical dentistry, ICR/PCR needs to be diagnosed as early and accurately as possible. However, concrete diagnostic criteria have yet to be established [4]. Therefore, ICR/PCR was recognized as an intractable disease by the Center for Intractable Disease Control in Japan in 2011 [5].

In the present study, we treated two patients with ICR that appeared to be caused by TMJ-OA in a reversible manner. One case was treated with an intraoral appliance only and followed up for 12 years. The other case was treated with intermaxillary fixation with anchor screws and mandibular traction. Both patients provided their consent to the publication of their cases in a journal.

## Case presentation

Case 1

Outline of the Patient

The age and sex of the patient was a 22-year-old female. The initial visit was in July 2006. Her complaints were difficulty opening the mouth and pain in front of the ears on both sides. There were no special remarks or systemic history. Rheumatoid factor quantitative test <3 (IU/m) = RA (-). There was no history of treatment related to female hormones such as the birth control pill.

Examination

**Figure 1:**
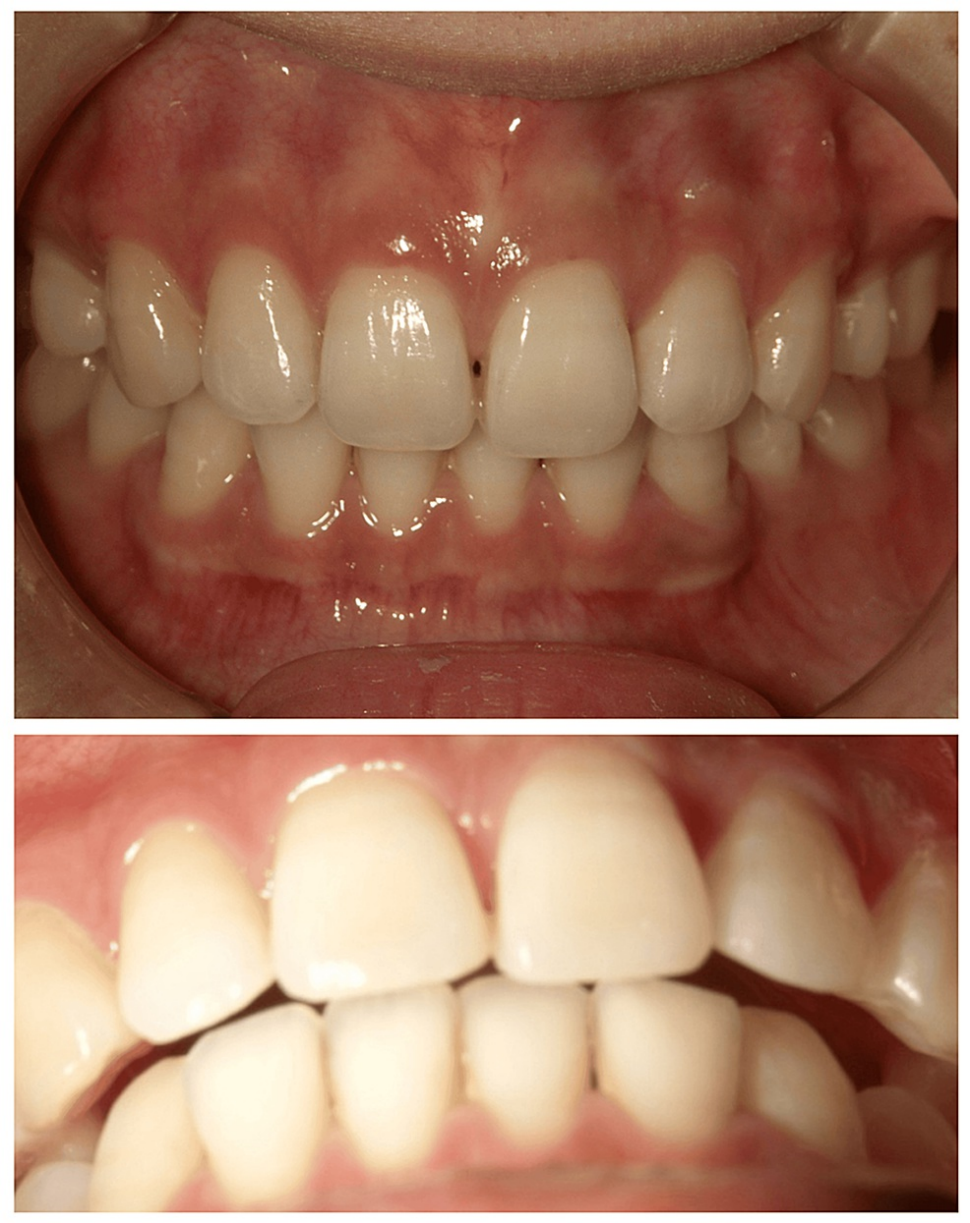
Photographs of the mouth at the initiation of treatment in Case 1

The patient completed orthodontic treatment with tooth extraction in 2003. An oral disturbance developed one year and six months ago. The patient then developed pain on opening the mouth and biting six months previously. There was no spontaneous pain in the muscles or TMJ at rest. However, dull pain occurred in the left and right TMJs at maximum opening. The occlusal condition showed occlusal contact only on the molars and an open bite on the anterior teeth. The intercuspal position contact relationship was unstable. There was a discrepancy between the midline of the upper and lower jaws, and the mandible deviated to the right side by approximately 2 mm (Figure 1). The occlusal pattern was bilaterally cuspid-guided. There was no abnormality in the position of the occlusal plane or the degree of deformity.

**Figure 2:**
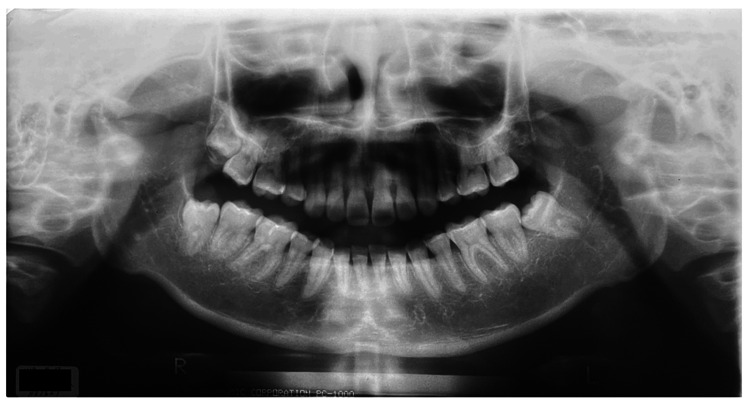
Panoramic radiography at the initiation of treatment in Case 1

An imaging examination using panoramic radiography revealed severe bone resorption in the right and left lateral mandibular heads (Figure 2). Cephalometric radiographs showed a Brachy face type. Dental 3D CT (3DCT, Asteion Super4: Toshiba, Japan) images showed that the height of the bilateral mandibular heads was reduced and the articular surfaces were eroded.

Clinical Diagnosis

The pathological diagnosis was ICR due to bilateral TMJ-OA (TMJ disorder type IV. This classification is published by the Japanese Society for Temporomandibular Joint).

Treatment Plan and Observations

In the initial visit, cognitive behavioral therapy was applied to teach the patient to become aware of her tooth-contacting habit (TCH) and daytime clenching and was performed in conjunction with instructions on the prevention of these habits.

At the beginning of treatment, when the patient was in pain, the non-steroidal anti-inflammatory analgesic, diclofenac sodium, was prescribed as an abortive medication.

**Figure 3:**
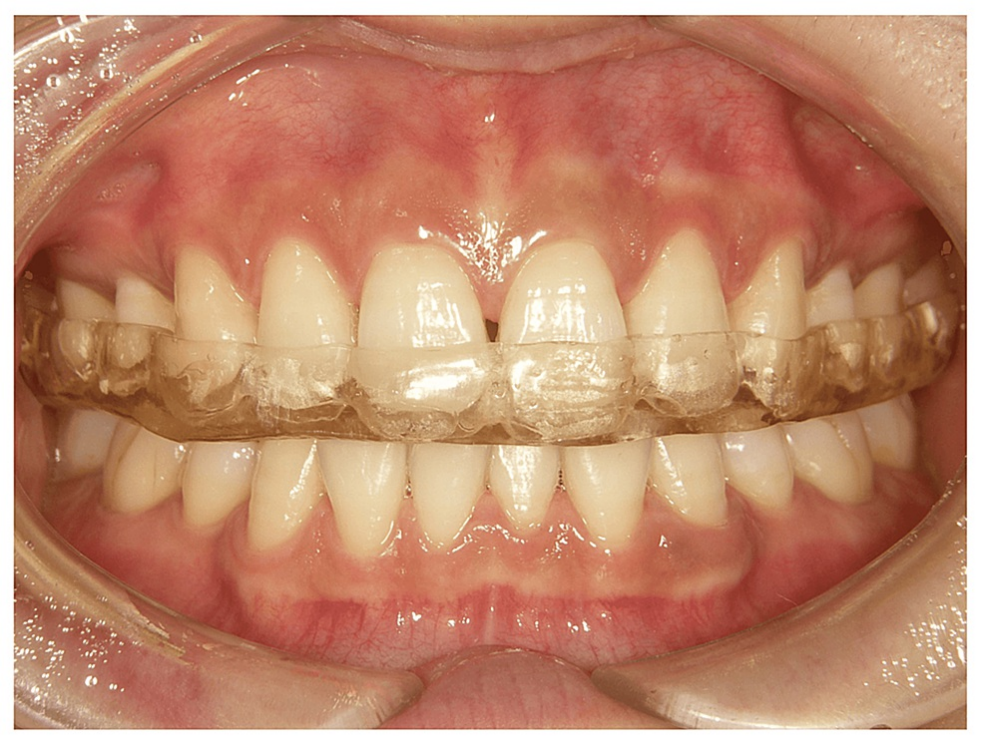
Photograph of the mouth during splint therapy

An anterior repositioning splint was fabricated. The period of use was limited to three months. The patient was then switched to a stabilization splint. One month after the introduction of the anterior repositioning splint, the patient’s symptoms had improved. After two months, motion pain during the opening disappeared and the extent of the opening increased (from 35 to 38 mm). After three months, the mandible moved anteriorly with no separation of the anterior teeth; therefore, a stabilization splint was fabricated in this mandibular position (Figure 3). After two months of wearing the stabilization splint, the patient was instructed to use the splint only at night because there was no recurrence of TMJ symptoms. After four months of treatment, the splint was temporarily discontinued because there was no recurrence of TMJ symptoms.

**Figure 4:**
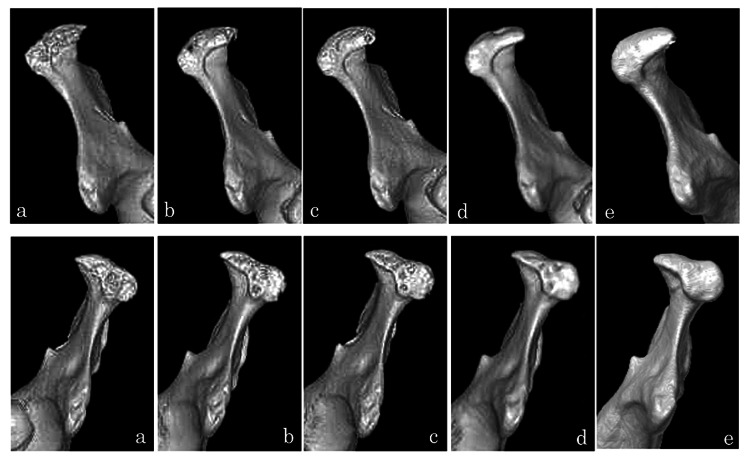
3DCT images of Case 1 Upper: right side, lower: left side, a: the initiation of treatment, b: the end of splint therapy, c: 1 year after splint therapy, d: 3 years after splint therapy t, e: 12 years after splint therapy

3DCT was taken at the end of splint therapy and in the follow-up (Figure 4). There were no signs of the recurrence of TMJ symptoms during this period, which confirmed that remodeling of the mandibular head was progressing. Erosion in the initial visit had improved and the mandibular position had changed. Conservative treatment was selected for the present case, and the patient’s condition was improved by the amelioration of erosion. Twelve years after the completion of splint therapy, the patient continued to show remodeling and further improvements in erosion. The occlusal relationship is stable and the patient does not have muscle tenderness or TMJ symptoms.

Case 2

Outline of the Patient

The age and sex of the patient was a 20-year-old female. The initial visit was in April 2021. Her complaints were unable to bite with the front teeth and the mouth was swollen and could not be closed. The patient requested further orthodontic treatment. There were no special remarks or systemic history. Rheumatoid factor quantitative test <3 (IU/m) = RA (-). There was no history of treatment related to female hormones, such as the birth control pill.

Examination

**Figure 5:**
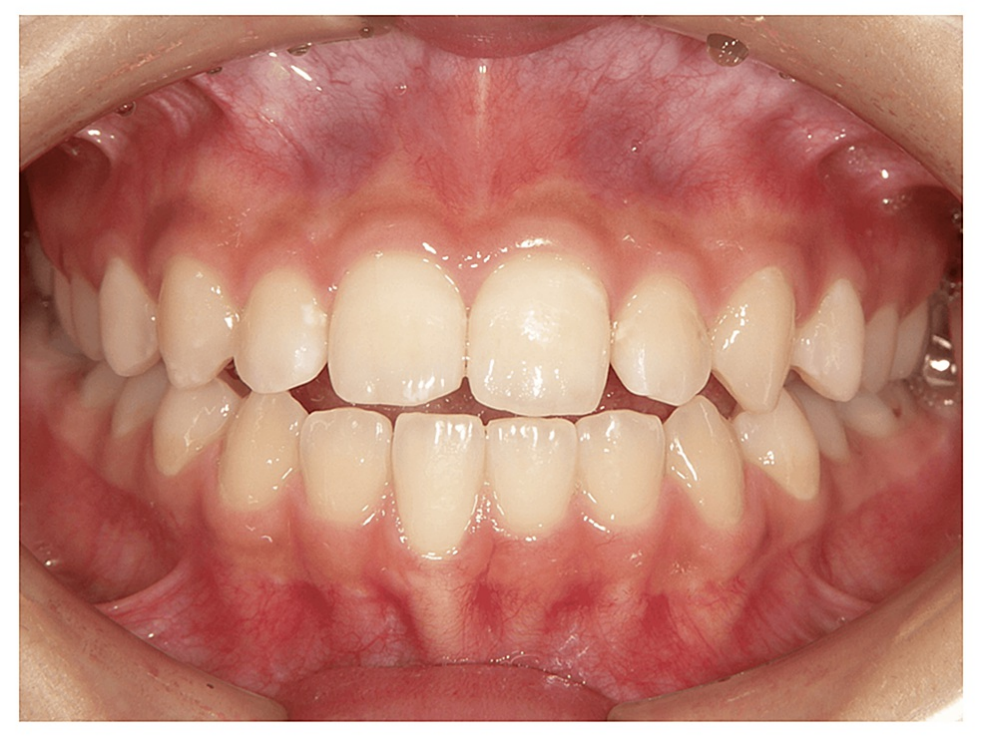
Photograph of the mouth at the initiation of treatment in Case 2

**Figure 6:**
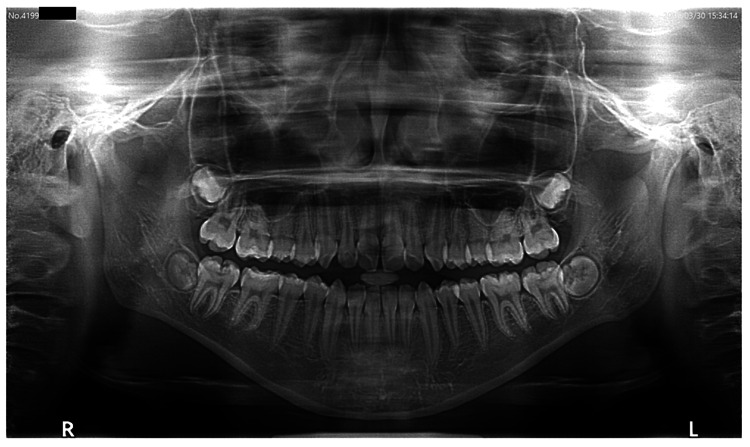
Panoramic radiography at the initiation of treatment in Case 2

Non-extraction orthodontic treatment was completed in 2018. The patient started treatment for dystopia at another hospital in 2019 and requested orthodontic re-treatment. There was no spontaneous pain in the muscles or TMJ at rest. There was a dull pain in the left and right TMJ at maximum opening. The opening distance was 39 mm. The contact relationship was unstable during oral mating. An open bite of the anterior teeth was observed (Figure 5). Panoramic radiographs revealed significant bone resorption in the right and left lateral mandibular heads (Figure 6). A Dolico face was diagnosed based on standard X-ray radiographs of the head.

**Figure 7:**
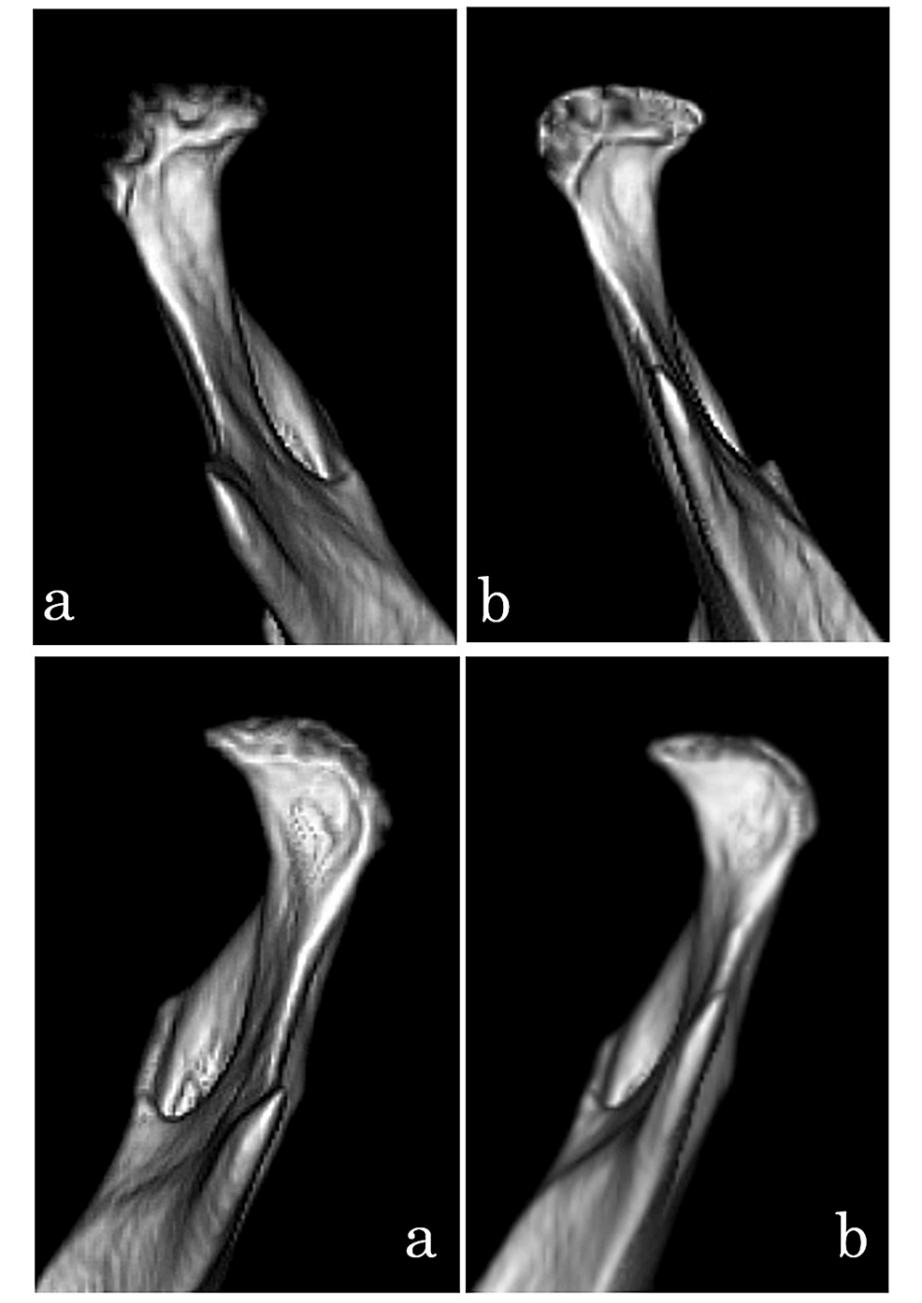
3DCT 3D-construction images of Case 2 Upper: right side, lower: left side, a: initial examination, b: 2 years after treatment

3DCT was taken at the end of splint therapy and in the follow-up (Figure 7). There were no signs of the recurrence of TMJ symptoms during this period, which confirmed that remodeling of the mandibular head was progressing.

Clinical Diagnosis

The pathological diagnosis was ICR due to bilateral TMJ-OA (TMJ disorder type IV).

Treatment Plan and Observations

In the initial visit, cognitive behavioral therapy was applied to teach the patient to become aware of TCH and daytime clenching and was performed in conjunction with instructions on the prevention of these habits.

**Figure 8:**
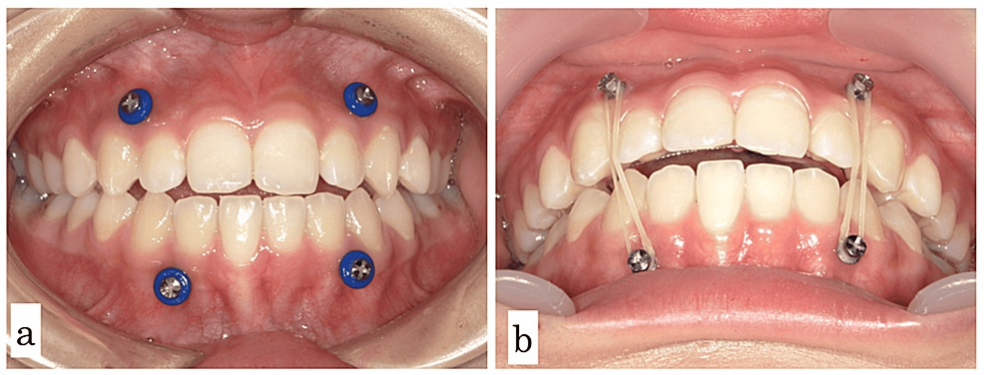
Photographs of the mouth during intermaxillary fixation a: after initial anchor screws, b: with traction elastics

Anchor screws (Dual Top Auto-Screw IIIJA, Proceed Japan) were placed between the maxillary bilateral lateral incisors and canines, and mandibular head traction was applied. Elastics were used, except during meals (Figure 8). Three months after the procedure, the overbite of the central incisor was 0 mm and the guide of the maxillary and maxillary central incisors was confirmed in the anterior position.

**Figure 9:**
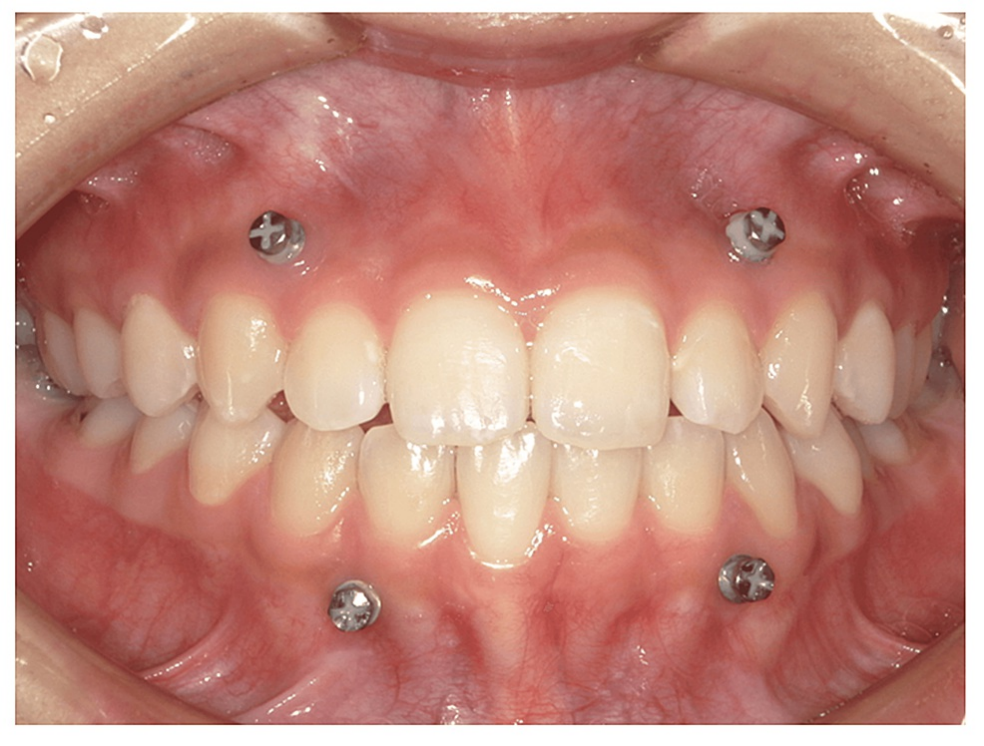
Photograph of the mouth two years after treatment in Case 2

Ten months after the procedure, the overbite of the central incisor was 0 mm, and anterior guiding contact was observed not only with the central incisor but also with the lateral incisor and canine. Elastics were subsequently used at night only. Two years after the procedure, the overbite was maintained at 1.0mm (Figure 9).

A resorption image of the mandibular head showed the amelioration of erosion, a change in the mandibular position, and an improvement in the open bite. Aggressive mandibular head traction therapy was performed instead of splint therapy, and an improvement in erosion and continuous remodeling were confirmed within only 10 months.

## Discussion

The pathophysiology of TMD has been organized by DC/TMD, and a highly accurate diagnosis by specialists and treatment based on this diagnosis is now available [6]. In addition to TMJ-OA, other factors suggested for the development of ICR/PCR include ischemic necrosis, infection [1,2,7], and the possibility of bone destruction of the mandibular head from direct or indirect lacerations due to contusions [8-10]. ICR/PCR is most frequently observed in females in their teens and early 20s, with a sex ratio of 1:9. Female hormones secreted from the uterus and other organs unique to women have been implicated in the development of ICR/PCR [11]. The pathologies of TMJ-OA and ICR were observed in the two cases in the present study, and TMJ-OA was presumed to be a factor in ICR. Tc99 scintigraphy could have been included in the definitive diagnosis. Our office where the patient was treated this time did not have scintigraphy equipment, so we did not include it in the examination. However, the high-resolution 3D CT images were able to observe the erosion of the temporomandibular joint in detail, and thus the diagnosis of ICR was made. The clinical implication of this report is to search for the establishment of a treatment for ICR or hints of such a treatment.

ICR refers to the sudden occurrence of mandibular head resorption without definitive or external factors, such as trauma or orthodontic treatment, and the underlying mechanism and disease concept was proposed by Arnett et al. in 1996 [8,9]. Although no clear differences in terminologies between ICR and PCR have been presented, the diagnosis of ICR in the present cases was based on bone resorption being absent in Case 1 for 12 years and progression being suppressed. In this condition, resorption of the mandibular head is accompanied by a posterior deviation of the entire mandible and premature contact of the posterior teeth. Masticatory and esthetic disorders resulting from an open bite of the anterior teeth develop. Along with abnormal occlusion, patients may have disorders such as masticatory muscle pain, arthralgia, and restricted mouth opening. A systematic review on condylar resorption [12] stated that stress on the mandibular head is a contributing factor and its removal is regarded as a treatment. TMJ, similar to other synovial joints in the body, is a load-bearing organ, and the mechanical load applied to the mandible head is essential for its growth, development, and maintenance [13]. Although the role of the articular disc remains unclear, the mandibular head may resorb over time in non-retrograde articular disc dislocation [14]. A recent study demonstrated that loading on the anterior surface of the condylar induced MMP-13, which is involved in articular cartilage degradation in the condylar head [15]. One possible treatment for ICR is surgical therapy for the condylar, but we did not choose this option. Because they have few blood vessels and are prone to scarring, condylar surgery is not recommended in much of the literature [8,9]. In our current study, we chose different treatments and mechanical stress release to the condylar. Both non-operative cases had good results. Based on the above, the policy of mechanical stress release seems to be correct. Furthermore, the treatment method with anchor screws is expected to shorten the treatment period.

Although there are currently no clear guidelines for the treatment of this condition [16], there are two main types of procedures to remove stress on the mandibular head: a technique to improve altered occlusion with a splint or other removable device [17,18], with many studies using stabilizing splints, and an orthognathic technique [19,20] that aims to improve occlusion fundamentally through full prosthetic treatment.

In principle, reversible and conservative measures are strongly recommended for treatment [17]. Stabilization splints may be used when excessive loading on TMJ due to unstable occlusion is suspected [18]. Case 1 selected conservative treatment, and after wearing a stabilization splint instead of an anterior position splint, her symptoms resolved and remodeling of the mandibular head was observed. The diagnosis of the onset mechanism in Case 1 is speculative because orthodontic treatment had been completed at the time of the initial visit; however, the occipital mating position was assumed to be stable at the end of orthodontic treatment 3 years earlier. Mechanical stress may have subsequently been triggered by factors that reduced the adaptive capacity or caused the jaw function system to over-function. Bone resorption of the mandibular head with backward rotation of the mandible and an open bite of the anterior teeth were assumed to have occurred. The patient was placed in a stable condyle position and splint therapy was used to stabilize the occlusal head in the newly obtained mandibular position. CT images 12 years later showed that remodeling of the mandibular head was still occurring after the completion of splint therapy. The release of stress on the mandibular head by guiding the patient to an appropriate mandibular position appeared to have exerted a positive effect.

**Figure 10:**
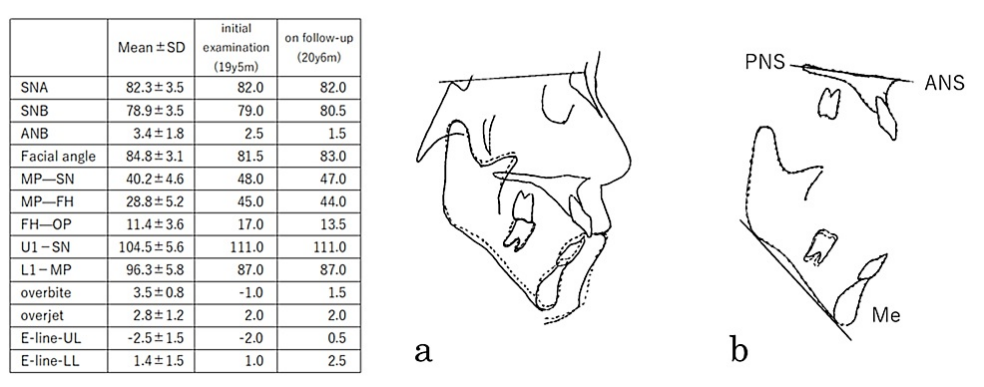
Results of a cephalometric analysis before and after treatment in Case 2 a: superimposition on the SN plane, b: Ricketts' superimposition method ANS: anterior nasal spine, PNS: posterior nasal spine, Me: menton Solid line: at the time of the first visit (19y 5m), dashed line: on follow-up (20y 6m)

The orthodontic results of the preoperative and postoperative analysis of Case 2 are shown (Figure 10). Mandibular pl. to SN went from 48.0° to 47.0°. In addition, Mandibular pl. to FH changed from 45.0° to 44.0° and the SNB angle changed from 79.0° to 80.5°. These suggest a counterclockwise shift of the mandible and a tightening of the occlusion. And the overbite was above zero. This implies an aggressive anterior bite.

On the other hand, traction of the upper and lower anterior teeth using rubber bands for active joint remodeling was previously shown to effectively shorten the treatment period [20], and intermaxillary fixation was used to remove stress on the mandibular head. We also consider it a reversible treatment and a promising option to shorten the treatment time. However, if teeth are used as a fixation source, tooth movement may occur with prolonged treatment. In Case 2, anchor screws were placed in the alveolar bone of the maxillary anterior teeth, and mandibular traction was performed, which resulted in remodeling of the mandibular head. Since the fixation source in this technique was anchor screw, tooth movement did not occur. In contrast to splint therapy, the release of forced mandibular head stress was possible. The patient will continue to be monitored for remodeling of the mandibular head.

## Conclusions

The two techniques presented herein were both performed to remove mechanical stress on the mandibular head. Both patients achieved good results with symptom resolution. In Case 1, after one year of an aggressive intervention with splinting, we observed continued remodeling of the mandibular cortical bone for 12 years. Case 2 was treated with an aggressive intervention with intermaxillary fixation therapy for 1 year.

## References

[1] Arnett GW, Gunson MJ (2013). Risk factors in the initiation of condylar resorption. Semin Orthod.

[2] Handelman CS, Greene CS (2013). Progressive/Idiopathic condylar resorption: an orthodontic perspective. Semin Orthod.

[3] Okeson JP (2019). Management of Temporomandibular Disorders and Occlusion. 8th edition. Mosby, St.Louis.

[4] Tanaka E (2023). Etiology and diagnosis for condylar resorption in growing adolescents. J Clin Med.

[5] (2023). Japan Intractable Diseases Infomation Center. Progressive condylar resorption (PCR) [In Japanese]. http://www.nanbyou.or.jp/entry/2401.

[6] Schiffman E, Ohrbach R, Truelove E (2014). Diagnostic criteria for temporomandibular disorders (DC/TMD) for clinical and research applications: recommendations of the international RDC/TMD consortium network and orofacial pain special interest group. J Oral Facial Pain Headache.

[7] Sarver DM, Janyavula S, Cron RQ (2013). Condylar degeneration and diseases - local and systemic etiologies. Semin Orthod.

[8] Arnett GW, Milam SB, Gottesman L (1996). Progressive mandibular retrusion—idiopathic condylar resorption. Part I. Am J Orthod Dentofacial Orthop.

[9] Arnett GW, Milam SB, Gottesman L (1996). Progressive mandibular retrusion—idiopathic condylar resorption. Part II. Am J Orthod Dentofacial Orthop.

[10] Choi J, Oh N, Kim IK (2005). A follow-up study of condyle fracture in children. Int J Oral Maxillofac Surg.

[11] Gunson MJ, Arnett GW, Formby B, Falzone C, Mathur R, Alexander C (2009). Oral contraceptive pill use and abnormal menstrual cycles in women with severe condylar resorption: a case for low serum 17β-estradiol as a major factor in progressive condylar resorption. Am J Orthod Dentofacial Orthop.

[12] Catherine Z, Breton P, Bouletreau P (2016). Condylar resorption after orthognathic surgery: a systematic review. Rev Stomatol Chir Maxillofac Chir Orale.

[13] Tanaka E, Koolstra JH (2008). Biomechanics of the temporomandibular joint. J Dent Res.

[14] Cortés D, Exss E, Marholz C, Millas R, Moncada G (2011). Association between disk position and degenerative bone changes of the temporomandibular joints: an imaging study in subjects with TMD. Cranio.

[15] Nogami S, Kataoka Y, Yamauchi K (2022). Condylar resorption following compressive mechanical stress in rabbit model - association of matrix metalloproteinases. In Vivo.

[16] Sansare K, Raghav M, Mallya SM, Karjodkar F (2015). Management-related outcomes and radiographic findings of idiopathic condylar resorption: a systematic review. Int J Oral Maxillofac Surg.

[17] (2023). AADR policy statement for temporomandibular disorders TMD. https://www.aadronline.org/i4a/pages/index.cfm.

[18] Wang XD, Zhang JN, Gan YH, Zhou YH (2015). Current understanding of pathogenesis and treatment of TMJ osteoarthritis. J Dent Res.

[19] Kau CH, Bejemir MP (2015). Application of virtual three-dimensional surgery planning in management of open bite with idiopathic condylar resorption. Ann Maxillofac Surg.

[20] Nitzan DW, Palla S (2017). “Closed reduction” principles can manage diverse conditions of temporomandibular joint vertical height loss: from displaced condylar fractures to idiopathic condylar resorption. J Oral Maxillofac Surg.

